# Huc-MSCs-derived exosomes attenuate inflammatory pain by regulating microglia pyroptosis and autophagy via the miR-146a-5p/TRAF6 axis

**DOI:** 10.1186/s12951-022-01522-6

**Published:** 2022-07-14

**Authors:** Tong Hua, Mei Yang, Honghao Song, Erliang Kong, Mengqiu Deng, Yongchang Li, Jian Li, Zhixiao Liu, Hailong Fu, Yue Wang, Hongbin Yuan

**Affiliations:** 1 Department of Anesthesiology, Changzheng Hospital, Naval Medical University, Shanghai, 200003 China; 2 Research Center of Developmental Biology, Department of Histology and Embryology, College of Basic Medicine, Naval Medical University, Shanghai, 200433 China; 3 Stem Cell and Regeneration Medicine Institute, Research Center of Translational Medicine, Naval Medical University, Shanghai, 200433 China

**Keywords:** Huc-MSCs-derived exosomes, Inflammatory pain, Microglia, Pyroptosis, Autophagy

## Abstract

**Background:**

Chronic inflammatory pain significantly reduces the quality of life and lacks effective interventions. In recent years, human umbilical cord mesenchymal stem cells (huc-MSCs)-derived exosomes have been used to relieve neuropathic pain and other inflammatory diseases as a promising cell-free therapeutic strategy. However, the therapeutic value of huc-MSCs-derived exosomes in complete Freund's adjuvant (CFA)-induced inflammatory pain remains to be confirmed. In this study, we investigated the therapeutic effect and related mechanisms of huc-MSCs-derived exosomes in a chronic inflammatory pain model.

**Methods:**

C57BL/6J male mice were used to establish a CFA-induced inflammatory pain model, and huc-MSCs-derived exosomes were intrathecally injected for 4 consecutive days. BV2 microglia cells were stimulated with lipopolysaccharide (LPS) plus adenosine triphosphate (ATP) to investigate the effect of huc-MSCs-derived exosomes on pyroptosis and autophagy. Bioinformatic analysis and rescue experiments were used to demonstrate the role of miR-146a-5p/ TRAF6 in regulating pyroptosis and autophagy. Western blotting, RT-qPCR, small interfering RNA and Yo-Pro-1 dye staining were performed to investigate the related mechanisms.

**Results:**

Huc-MSCs-derived exosomes alleviated mechanical allodynia and thermal hyperalgesia in CFA-induced inflammatory pain. Furthermore, huc-MSCs-derived exosomes attenuated neuroinflammation by increasing the expression of autophagy-related proteins (LC3-II and beclin1) and inhibiting the activation of NLRP3 inflammasomes in the spinal cord dorsal horn. In vitro, NLRP3 inflammasome components (NLRP3, caspase1-p20, ASC) and gasdermin D (GSDMD-F, GSDMD-N) were inhibited in BV2 cells pretreated with huc-MSCs-derived exosomes. Western blot and Yo-Pro-1 dye staining demonstrated that 3-MA, an autophagy inhibitor, weakened the protective effect of huc-MSCs-derived exosomes on BV2 cell pyroptosis. Importantly, huc-MSCs-derived exosomes transfected with miR-146a-5p mimic promoted autophagy and inhibited BV2 cell pyroptosis. TRAF6, as a target gene of miR-146a-5p, was knocked down via small-interfering RNA, which increased pyroptosis and inhibited autophagy.

**Conclusion:**

Huc-MSCs-derived exosomes attenuated inflammatory pain via miR-146a-5p/TRAF6, which increased the level of autophagy and inhibited pyroptosis.

**Graphical Abstract:**

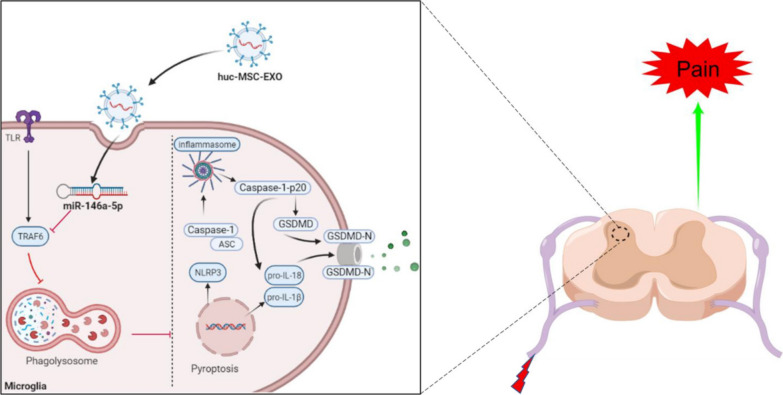

**Supplementary Information:**

The online version contains supplementary material available at 10.1186/s12951-022-01522-6.

## Background

Chronic pain, such as chronic inflammatory pain, is one of the most common and complex health problems, which causes insomnia, depression and increases suicide occurrence [1]. In addition, chronic pain brings about huge social and economic burdens [2]. However, the mechanism of chronic pain is still not completely understood, leading to a lack of effective treatments. Available evidence in the past few years suggests that neuroinflammation plays a predominant role in the pathological process of chronic pain [3, 4]. Opioids and non-steroidal anti-inflammatory drugs (NSAIDs) are most commonly used to alleviate chronic pain resulting from neuroinflammation [5]. However, continuously using opioids increases the risk of serious adverse effects, such as drug addiction, opioid tolerance, and opioid-induced hyperalgesia [6]. On the other hand, opioids and NSAIDs cannot completely inhibit the generation of chronic pain. Thus, it is imperative to pay more attention to exploring the mechanisms of chronic inflammatory pain, and develop an effective therapeutic strategy.

Microglia play a double-edged sword role in protecting against stimulation resulting from tissue damage or pathogen infection. An excessive inflammatory response, especially secreting abundant proinflammatory cytokines, eventually leads to neuroinflammation [7]. Microglia pyroptosis is a newly discovered inflammatory cell death via inflammasomes associated with immune and inflammation-related disorders in the central nervous system, covering spinal cord injury and depression [8–10]. NLRP3 inflammasome is one classic member of inflammasomes which is the most studied molecules in chronic pain [11, 12]. The main components of NLRP3 inflammasome include pattern recognition receptor NLRP3, adaptor protein apoptosis-associated speck-like protein (ASC) and pro-caspase-1 enzyme [13]. Once NLRP3 inflammasome is activated by a diversity of stimuli and ligands, the assembly of NLRP3 with ASC into the inflammasome complex is triggered. The activation of NLRP3 and caspase-1 cleaves pro-IL-1β and pro-IL-18, ultimately increasing the secretion of mature IL-1β and IL-18 [13]. Moreover, NLRP3 inflammasome is closely related to pyroptosis, via cleaving GSDMD into its active form, GSDMD-N, which forms pores in the cell membranes [14]. Autophagy is an evolutionarily conserved biological process that eliminates misfolded proteins or ligands and influences immune inflammation and chronic pain [15]. Studies have demonstrated that autophagy can inhibit the activation of NLRP3 inflammasome and the secretion of mature IL-1β and IL-18 via eliminating hazardous substances such as damaged mitochondria [16]. Impaired autophagic flow aggravates inflammation by increasing the activation of NLRP3 and GSDMD-N-induced pyroptosis. Our previous research found that impaired autophagic flux aggravated neuropathic pain by increasing the levels of neuroinflammation and ROS [15]. However, it is still unclear whether autophagy influences pyroptosis and neuroinflammation in chronic inflammatory pain.

In recent decades, MSCs have remained a research hotspot. MSCs have immunomodulatory advantages, inhibiting inflammation and promoting tissue repair. It is the main mechanism by which huc-MSCs exert biological functions via paracrine action [17]. It has been reported that MSCs have optimistic treatment effects on neuropathic pain, bone cancer pain and other chronic pain [18, 19]. However, there are some limitations to use MSCs to alleviate chronic pain. First, it is inconvenient to process and preserve MSCs. Second, only a few transplanted MSCs can migrate to the target tissues and have a low survival rate in an inflammatory environment. Third, there are some potential risks of MSCs therapies, such as pulmonary thrombosis or endogenous tumour formation. Among the MSCs paracrine factors, exosomes have prominent therapeutic potential in a variety of inflammatory diseases, such as Parkinson’s disease, inflammatory bowel disease, spinal cord injury and chronic pain [20–22]. As MSCs-derived extracellular vesicles, exosomes are bilayer lipid nanoparticles with a diameter of 30–150 nm. Exosomes are considered to mediate cell-to-cell interactions or information exchange via transferring bioactive cargos, such as proteins, microRNAs, DNA and lipids [23]. A previous study found that single or continuous intrathecal infusion of huc-MSCs-derived exosomes achieved remarkable treatment effects on nerve injury induced acute or chronic pain [22]. Although the analgesic effects may be related to inhibiting the activation of neurons and glial cells, the definite mechanism remains elusive. Recently, another study showed that huc-MSCs-derived exosomes protected microglia from pyroptosis by enhancing mitophagy [24]. Another study also showed that huc-MSCs-derived exosomes protected against DSS-induced colitis via inhibiting the activation of NLRP3 inflammasomes and subsequent pyroptosis [13]. Therefore, we hypothesized that huc-MSCs-derived exosomes may attenuate CFA-induced chronic inflammatory pain via enhancing autophagy and inhibiting pyroptosis.

In the present study, our research objective was to demonstrate the analgesic effect of huc-MSCs-derived exosomes on CFA-induced chronic inflammatory pain. Our findings indicate that huc-MSCs-derived exosomes attenuate chronic inflammatory pain by inhibiting GSDMD-induced microglia pyroptosis through promoting autophagy. miR-146a-5p enriched in huc-MSCs-derived exosomes plays an important role in analgesic effects by negatively regulating TRAF6.

## Methods

### Experimental animals

Wild-type C57BL/6 J male mice (7–8 weeks old) were purchased from Animal Experiments Center of Naval Medical University (NMU) (Shanghai, China). All animals reached the specific pathogen free (SPF) standard. Mice were housed in closed circulation ventilation cages, with no more than 5 animals in a single cage. The animals were maintained at constant temperature (23 ± 1 °C) and 55% humidity under a 12-12 h light–dark cycle, with a sufficient supply of food and water. All animal experiments were performed in accordance with the Scientific Investigation Committee of NMU, and the guidelines of the Ethics Committee of the International Pain Research Association were followed. The animal protocols were approved by the Animal Committee of changzheng hospital.

### CFA-induced inflammatory pain model and drugs or huc-MSCs-derived exosomes administration

A chronic inflammatory pain model was induced by CFA (F5881, Sigma-Aldrich, GER), as previously described [25]. In brief, 20 µL of CFA was subcutaneously injected into the left hind paw under anesthesia (pentobarbital sodium, 50 mg/kg, injected intraperitoneally). Mice randomly received a daily intrathecal injection of huc-MSCs-derived exosomes (5 µg in 5 µL) or saline starting from the same day for 4 consecutive days. Another group of mice receiving saline injection to the hind paw as well as intrathecally were included as an additional control. In separate experiments, mice received either 3-MA (5 µg/5 µL) (HY-19312, MCE, China) or rapamycin (RAP, 5 µg/5 µL) (HY-10219, MCE, China) intrathecally at 1 h before CFA injection.

### Cell culture and huc-MSCs-derived exosomes purification and identification

BV2, a microglia cell line, was purchased from the Chinese Type Culture Collection. BV2 cell line was cultured in high-sugar DMEM (8121513, Gibco, USA) containing 10% fetal bovine serum (10099141, Gibco), 100 U/mL penicillin, and 100 µg/mL streptomycin (15070063, Gibco) and grown in a condition of 5% CO2 and 95% humidity at 37 °C. Huc-MSCs were obtained from the department of histology and embryology, NMU. Huc-MSCs were cultured in cell-farm medium (Wobisheng, China) containing 10% exosome-free fetal bovine serum (10099141, Gibco, USA), and grown in a condition of 5% CO2 and 95% humidity at 37 °C. Huc-MSCs were used at passage 2 to 6 in exosome-free medium, then the culture supernatant was collected and stored in − 80 °C. Sufficient culture supernatant was centrifuged for 10 min at 300 × g, 10 min at 2000 × *g*, and 30 min at 10,000 × *g* at 4 °C to eliminate foreign substances. The supernatant was collected and centrifuged twice for 70 min at 100,000 × *g* at 4 °C in new tubes suitable for ultracentrifuge rotors. Then, the new supernatant was removed, and the remaining deposit was resuspended in PBS and stored at − 20 °C [26]. The concentration was measured by the BCA method (ZJ101, Epizyme, China). The exosomal surface marker proteins were tested by western blot. Transmission electron microscopy (TEM) was used to observe the morphology of huc-MSCs-derived exosomes. The diameter of exosomes was measured by a NanoSight detector [27].

### Behavioral tests

#### Mechanical allodynia

First, mice were put into the animal behavior test room in a transparent plastic box adapting for 30 min on a wire mesh platform. Their paw mechanical withdrawal thresholds (PWTs) were measured using the electronic von Frey test (IITC/Life Science, USA) with an appropriate probe that stimulated the hind paw plantar with incremental forces. When a force induced a paw withdrawal response, such as shrinking their feet, escaping or licking their feet, the value was recorded on the machine. Each animal was measured 3 times at an interval of 10 min and the average value of the three duplicate tests was recorded as the PWT. The baseline value of PWT was obtained 1 day before CFA injection, and the PWT was tested on days 1, 3, and 7 after CFA, drugs or huc-MSCs-derived exosomes administration.

#### Thermal hyperalgesia

Mice were adapted to the same environment in a transparent plastic box for 30 min on a glass surface before their thermal withdrawal latency (TWL) was evaluated. A Hargreaves radiant heat apparatus (IITC/Life Science, USA) was used to stimulate the hind paw until paw withdrawal responses were induced. To avoid tissue damage, a cut-off value of 25 s was set. Each animal was measured 3 times at an interval of 10 min and the average value of the three duplicate tests was recorded as the TWL [15]. The baseline value of TWL was obtained 1 day before constructing the model, and the TWL was tested on days 1, 3, and 7 after CFA, drugs or huc-MSCs-derived exosomes administration.

### Cell viability CCK-8 assay

The influence of huc-MSCs-derived exosomes on the viability of BV2 cells was detected via CCK-8 kit (HY-K0301, MCE, China). According to the instructions, 1000 BV2 cells were seeded in each well of a 96-well plate and treated with different concentrations of huc-MSCs-derived exosomes (0, 10, 50, 100 mg/mL) for 24 h. Then, 100 µL of complete medium mixed with 10 µL of CCK-8 reagent was added to each well for 1–2 h. In the end, the absorbance was measured using infinite M200 PRO (TECAN) at 450 nm.

### Western blot analysis

Spinal cord tissues or different groups of BV2 cells were lysed in RIPA lysis buffer containing a protease and phosphate inhibitor (PC101, Epizyme, China). BCA kit was used to measure protein concentrations in different groups. The same amount of protein (30 µg) was separated on 10% or 12.5% SDS PAGE and was wet-electrotransferred onto 0.2 µm polyvinylidene fluoride (PVDF) membranes (Millipore, USA). Protein free rapid blocking buffer (PS108, Epizyme, China) was used to block the membrane for 45 min at room temperature on a rocker. Then, the PVDF membranes were incubated with different primary antibodies for 16 h at 4℃. The primary antibodies included anti-calnexin (1:1000, ab133615, Abcam), anti-CD44 (1:1000, ab243894, Abcam), anti-CD9 (1:1000, ab236630, Abcam), anti-CD63 (1:1000, ab134045, Abcam), anti-GAPDH (1:1000, 5174, CST), anti-iba1 (1:1000, ab178846, Abcam), anti-NLRP3 (1:1000, 15101, CST), anti-GSDMD-F (1:1000, 39754, CST), anti-GSDMD-N (1:1000, 39754, CST), and anti-mature IL-1β (1:1000, sc-12742, Santa Cruz), anti-caspase-1-p20 (1:1000, sc-398715, Santa Cruz), anti-ASC (1:1000, sc-514414, Santa Cruz), anti-beclin1 (1:1000, 3495, CST), anti-P62 (1:1000, 39786, CST), anti-LC3 (1:1000, 4108, CST), anti-TRAF6 (1:1000, A0973, ABclonal). On the next day, different membranes were washed with TBST for 30 min, and then incubated with secondary antibody at room temperature for 1–2 h. Protein bands were measured using enhanced chemiluminescence (ECL) reagent (Simuwu, China) and imaged with a gel imaging system (Tanon, China). ImageJ software (NIH, USA) was used for quantitative analysis.

### Quantitative real-time polymerase chain reaction (RT-qPCR)

Total RNA was extracted from spinal cords or cells using TRIzol reagent (R401-01, Vezyme, China). The RNA was reverse-transcribed to cDNA with a reverse transcription kit (YESEN, China). The RT-qPCR assay was used to detect the expression of target genes using a kit (YESEN, China). The RT-qPCR of miRNA was tested by using Bulge-LoopTM miRNA qRT-PCR Starter Kit (Ribo, China). The cycle threshold (Ct) values of target genes were collected and normalized to that of β-actin, and the fold change in gene expression was calculated with the 2^−ΔΔCT^ method. The primer sequences are shown in Table 1.

**Table 1:** Primer sequences for RT-PCR

Genes	Primer sequence
β-actin	Forward: 5′-GGCTGTATTCCCCTCCATCG-3′ Reverse: 5′-CCAGTTGGTAACAATGCCATGT-3′
IL-1β	Forward: 5′-AGAGCCCATC CTCTGTGACT-3′ Reverse: 5′-GCTCATATGG GTCCGACAGC-3′
IL-18	Forward: 5′-GACTCTTGCGTCAACTTCAAGG-3′ Reverse: 5′-CAGGCTGTCTTTTGTCAACGA-3′
TARF6	Forward: 5′AAAGCGAGAGATTCTTTCCCTG-3′ Reverse: 5′-ACTGGGGACAATTCACTAGAGC-3′

### Small-interfering RNA, miRNA mimic and inhibitor transfection

Huc-MSCs were incubated in 125 cm^2^ cell culture plates and transfected with miRNA mimic and inhibitor when the cell density reached approximately 30–50% confluency. The miR-146a-5p mimic, miR-146a-5p inhibitor and a negative control were directly transfected via riboFECT™ CP (Ribo, China) for 48 h. The transfected huc-MSCs were further incubated in cell-farm medium containing 10% exosome-free fetal bovine serum, and the culture supernatant was collected. Different groups of exosomes were extracted from the culture supernatant using ultracentrifugation. Three TRAF6 small-interfering RNAs (siRNA, Ribo, China) and a negative control (si-NC) were used to transfect BV2 cells at a confluency of 70–90% using Lipofectamine™ 3000 (Thermo, USA) according to the manufacturer’s instructions. After 48 h, BV2 cells were stimulated with LPS (1 µg/mL, Sigma-Aldrich, GER) for 24 h and ATP (5 mM, Sigma-Aldrich, GER) for 30 min.

### Yo-Pro-1 dye uptake

Yo-Pro-1 (Beyotime, China) is a small molecular dye which is membrane impermeable but pyroptosis-pole permeable. The method used in the dye uptake assay was conducted as previously described [9]. We used the dye (0.2 mM) to observe BV2 cells pyroptosis. BV2 cells were pretreated with 100 mg/mL exosomes, 3-MA (5 mM) for 1 h, which were then stimulated with LPS (1 µg/mL) for 24 h and then ATP (5 mM) for 30 min. 0.1% Triton which can destroy cell membrane was used to be a positive control. Hoechst 33,258 (Beyotime, China) was used to stain nucleus, indicating the total cells. The observation and images were obtained by fluorescence microscope (Zeiss, Germany).

### Enzyme-linked immunosorbent assay (ELISA)

BV2 cell supernatant samples were collected from different groups, including exosome-pretreated cells and LPS plus ATP-stimulated cells. Then, the supernatant samples were centrifuged for 10 min at 3000 × *g* at 4 °C and the deposit was removed. Collecting the new culture supernatant and measured the concentration of IL-1β with IL-1β ELISA kits (mlBio, China) according to the manufacturer’s instructions.

### Autophagic flux measurements via AdmCherry-GFP-LC3

We used mCherry green fluorescent protein (GFP)-tagged LC3 adenovirus (AdmCherry-GFP-LC3) (Hanbio, China) to observe the change in autophagy as previously reported [28]. Green fluorescence will be quenched when GFP enters the acidic environment in lysosomal. On the other hand, mCherry is stable and insensitive to the acidic environment. Finally, the autophagic flux can be measured by observing autophagosomes (yellow) and autolysosomes (red). The BV2 cells were transfected with AdmCherry-GFP-LC3 (MOI = 30) for 48 h. Then, the BV2 cells transfected with si-NC or si-TRAF6 were stimulated with LPS plus ATP for 24 h.

### Immunofluorescence

After anesthetization with pentobarbital sodium, different groups of mice were intracardially injected with PBS until the livers turned pale. Then, 4% ice-cold PFA was injected into the mice until their bodies became stiff. The lumbar enlargements of spinal cord tissues were dissected and fixed in PFA for 8 h, and 25% sucrose solution was used to dehydrate the tissues until they sunk to the bottom of the tube. The tissues were embedded in O.T.C tissue freezing medium (SAKURA, USA) and sectioned into 20 µm-thick slices, as previously described [29]. Different groups of sections were incubated with iba1 antibody (1:200, Abcam) at room temperature overnight. On the next day, the sections were incubated with secondary antibodies at room temperature for 1 h. Observation and photographic documentation were conducted using a fluorescence microscope (EclipseE600, Nikon, Japan).

### Data analysis

Data are shown as the mean ± standard error of the mean (SEM), and results were analysed using GraphPad Prism 8 software (GraphPad Software, USA). A two-tailed Student's t-test was used for statistical analysis of two groups, and one-way analysis of variance was used for multiple comparisons with Bonferroni post hoc analysis. Two-way ANOVA and two-way repeated-measures ANOVA followed by Dunnett’s post hoc test was used in behavioral test data. Differences were considered statistically significant if the *P* value was < 0.05.

## Results

### Characterization of huc-MSCs-derived exosomes

**Fig. 1:**
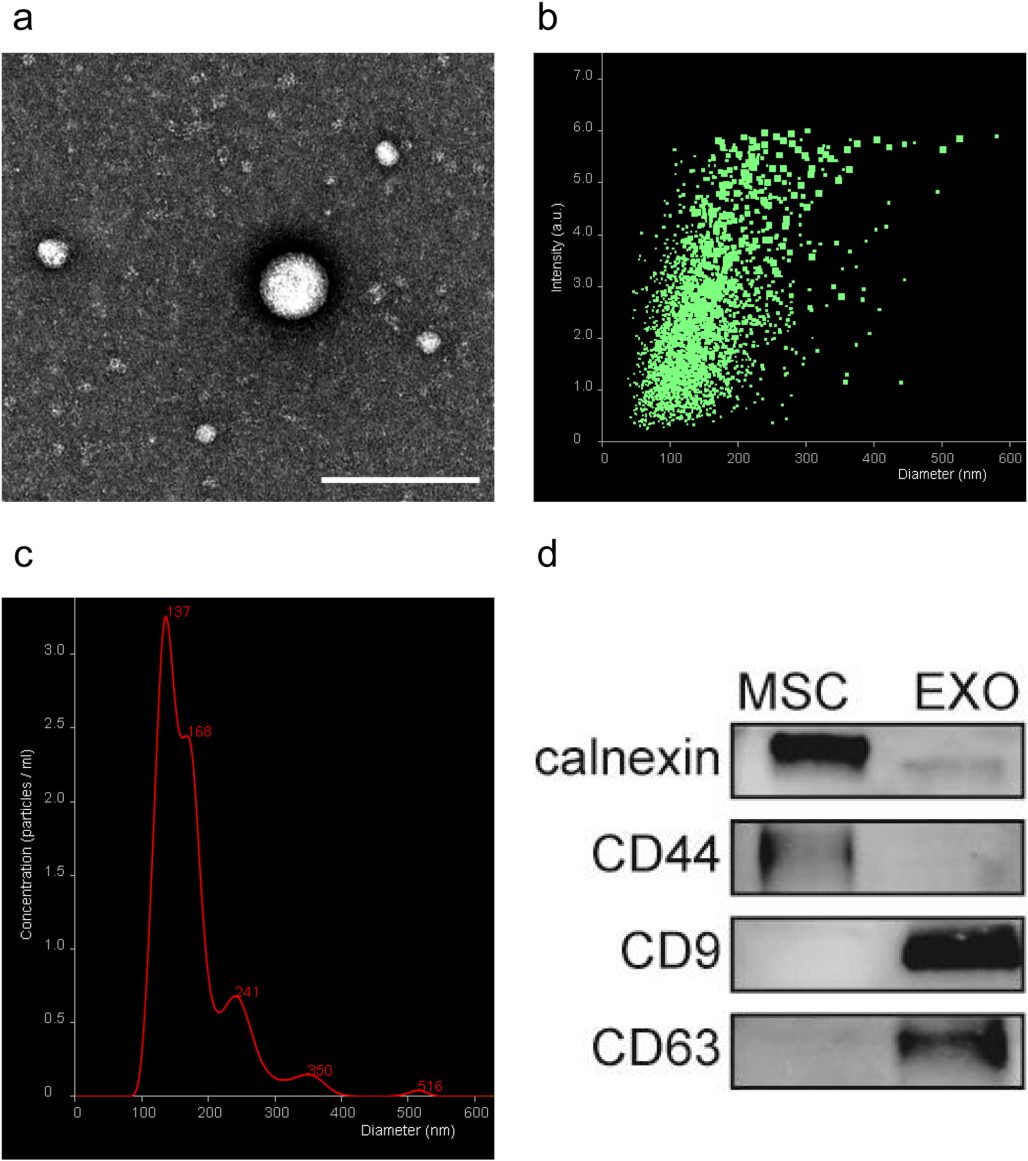
Identification of huc-MSCs-derived exosomes. **a** Morphological photos of typical huc-MSCs-derived exosomes under TEM, bar = 200 nm. **b** A nanoparticle tracking analyser detected the relative intensity of exosomes. **c** Nanoparticle tracking analysis of huc-MSCs-derived exosomes diameters. **d** Western blot analysis of exosome-related surface markers, including CD9 and CD63, and huc-MSCs-related markers calnexin and CD44

Although there is no unified standard for the identification of exosomes, the methods commonly used to observe the identity and purity of exosomes include western blot, TEM, and nanoparticle tracking analysis (NTA). Huc-MSCs-derived exosomes exhibited a typical double concave disc-like shape which was approximately 100 nm in size via the observation of morphology by TEM (Fig. 1a). NTA was used to measure the diameter and relative intensity of huc-MSCs-derived exosomes. The obtained results showed that the average diameter of huc-MSCs-derived exosomes was approximately 140 nm (Fig. 1b, c). Western blot results showed that the exosomal surface marker proteins CD9 and CD63 were all expressed but negative for the huc-MSC marker proteins calnexin and CD44 (Fig. 1d).

### Huc-MSCs-derived exosomes attenuate CFA-induced inflammatory pain and neuroinflammation

**Fig. 2:**
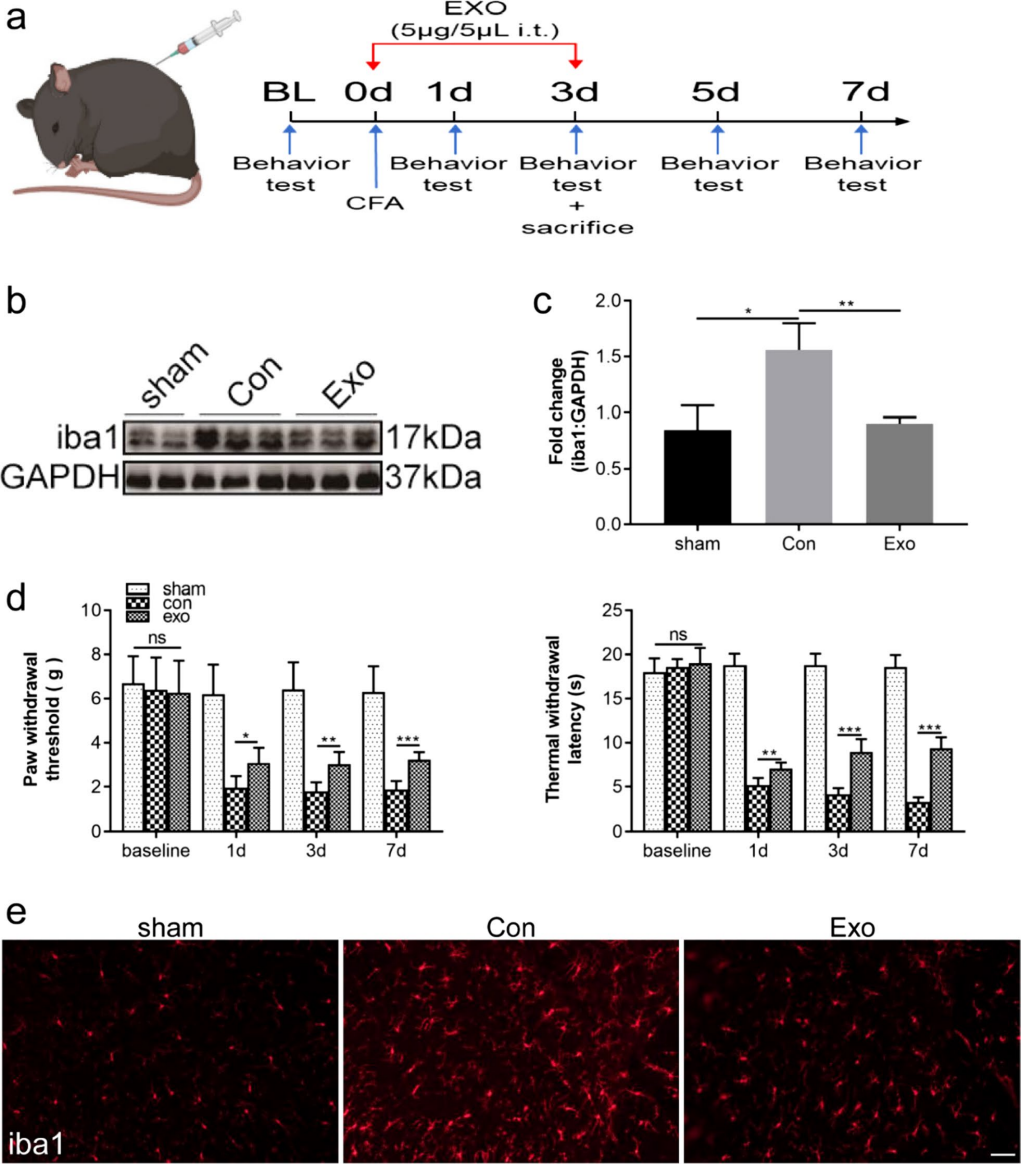
Huc-MSCs-derived exosomes attenuated CFA-induced inflammatory pain and neuroinflammation. **a** Mice were administered saline or exosomes via intrathecal (i.t.) injection for four successive days, followed by behavior testing or sacrifice at the indicated time points. **b**, **c** The protein level of iba1 was tested by western blot in the sham, Con and Exo groups. **d** Immunofluorescence detected the microglia proliferation in the sham, Con and Exo groups, bar = 50 µm. **e** Compared with the con group, exosomes increased paw withdrawal threshold and thermal withdrawal latency. Data are presented as mean ± standard deviation (SD). (*P < 0.05, **P < 0.01, ***P < 0.001)

CFA-induced inflammatory pain is a classic chronic inflammatory pain model [30], and this model was used for our study. We designed a study protocol which aimed to investigate the protective effects of huc-MSCs-derived exosomes on attenuating CFA-induced inflammatory pain and neuroinflammation (Fig. 2a). On the 3^rd^ day after CFA injection, huc-MSCs-derived exosomes inhibited the abnormal activation and proliferation of microglia in the left spinal cord dorsal horn. Compared with the control group, the expression of iba1, a microglial marker, was significantly inhibited via huc-MSCs-derived exosomes intrathecal (i.t.) injection for four successive days (Fig. 2b, c). Immunofluorescence analysis also demonstrated that iba-1 expression was decreased in the group treated with huc-MSCs-derived exosomes (Fig. 2d). The results also revealed that the PWT and TWL were decreased from the 1^st^ day and persistently maintained until day 7 after CFA injection compared with the sham group. Huc-MSCs-derived exosomes were intrathecally injected for four successive days from 1 h before modelling, and the PWT and TWL were increased on day 1, 3, 7 compared with the control group. The results showed that huc-MSCs-derived exosomes alleviated mechanical allodynia or thermal hyperalgesia in CFA-induced inflammatory pain (Fig. 2e).

### Huc-MSCs-derived exosomes alleviate the activation of NLRP3 inflammasome and GSDMD-induced pyroptosis in LPS plus ATP-treated BV2 cells

**Fig. 3:**
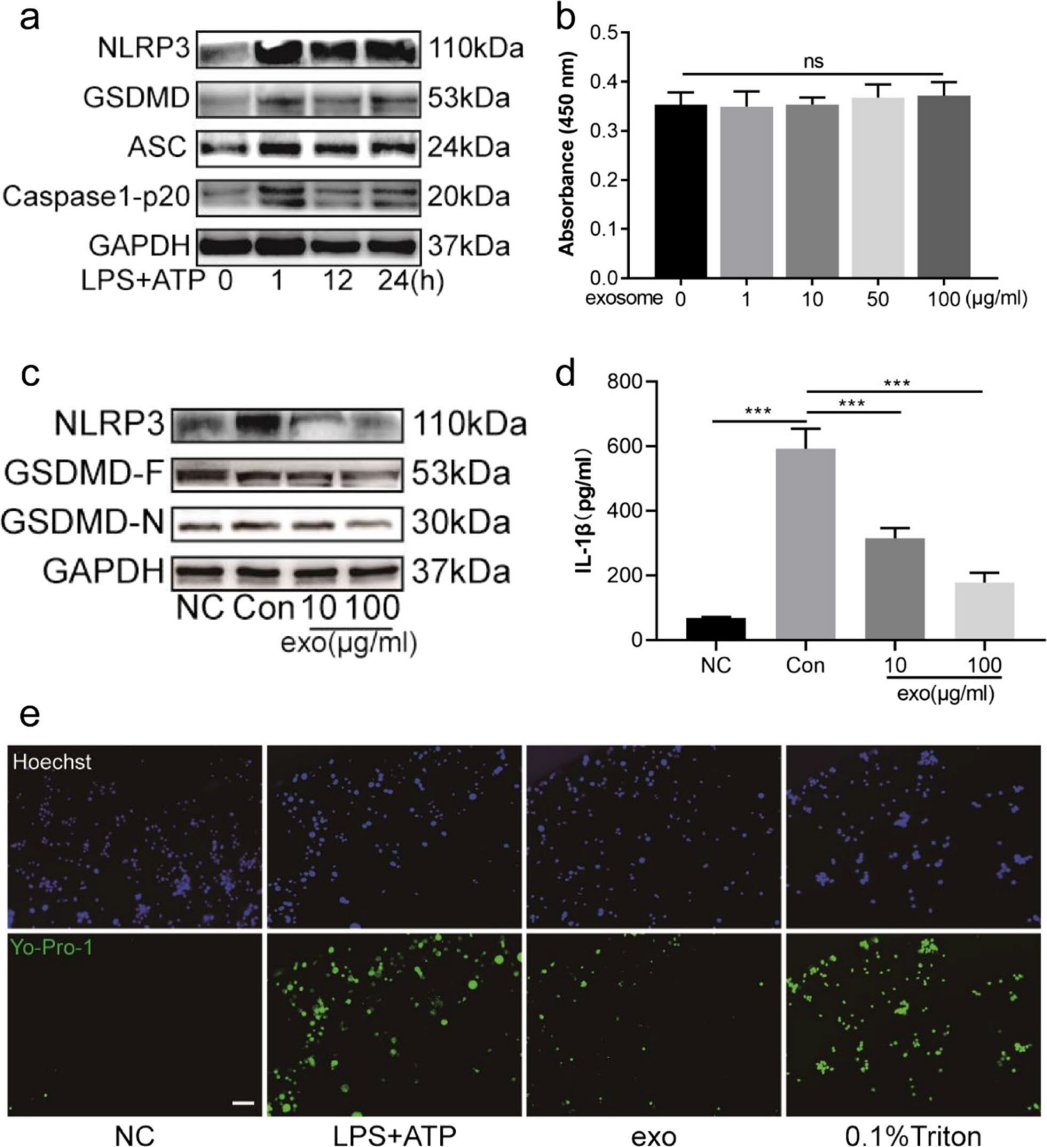
Huc-MSCs-derived exosomes inhibited BV2 cells pyroptosis. **a** BV2 cells were administered LPS (1 µg/ml) for 1 h, 12 h, 24 h and ATP (5 mM) for 30 min, and NLRP3, GSDND, ASC and caspase-1-p20 were tested by western blot at the indicated time points. **b** CCK-8 assay was used to assess the effect of huc-MSCs-derived exosomes (1, 10, 50, 100 µg/ml) on BV2 cell viability, and ns indicates no significance. **c** Compared with con group, the protein levels of NLRP3, GSDMD-F, and GSDMD-N were decreased in BV2 cells pretreated with 10 and 100 µg/ml exosomes. **d** ELISA was used to test IL-1β in supernatants from different groups. **e** Different groups of BV2 cells were stained with Yo-Pro-1 (green), which is membrane impermeable but pyroptosis-pole permeable dye. Hoechst (blue) was used to stain nucleus. BV2 cells treated with 0.1% Triton were used to be the positive control group, bar = 50 µm. Data are presented as the mean ± standard deviation (SD). (***P < 0.001)

Microglia play a prominent role in aggravating chronic pain by enhancing the release of proinflammatory cytokines. NLRP3 inflammasome activation is one of the most important mediators [31]. On the other hand, NLRP3 inflammasome activation can increase and cleave GSDMD, resulting in the generation of GSDMD-N, which forms pores in the cell membrane. Our results showed that the expression levels of NLRP3, GSDMD, ASC and caspase1-p20 were increased in LPS and ATP-treated BV2 cells at different indicated time points (Fig. 3a). Meanwhile, a CCK-8 kit was used to determine the effect of different concentrations of huc-MSCs-derived exosomes on the viability of BV2 cells. We chose 10 mg/mL and 100 mg/mL huc-MSCs-derived exosomes to pretreat BV2 cells according to the result showing that 100 mg/mL had no cytotoxicity (Fig. 3b). Western blot analysis demonstrated that huc-MSCs-derived exosomes suppressed the expression of NLRP3 inflammasome-related proteins and GSDMD-F, GSDMD-N in a concentration-dependent manner (Fig. 3c). To investigate the protective value of huc-MSCs-derived exosomes in inhibiting the maturation and release of IL-1β, ELISA was used (Fig. 3d). GSDMD-N forms pores with an average inner diameter of approximately 13 nm, through which mature IL-1β and IL-18 are released [9]. Thus, Yo-Pro-1, a small membrane impermeable but pyroptosis-pole permeable dye, was used to visually observe BV2 cells pyroptosis as reported [32]. Our results showed that huc-MSCs-derived exosomes decreased the uptake of Yo-Pro-1 dye compared with the LPS + ATP group (Fig. 3e). Thus, these results demonstrated that huc-MSCs-derived exosomes alleviated the activation of NLRP3 inflammasome and GSDMD-induced pyroptosis in LPS and ATP-treated BV2 cells.

### Huc-MSCs-derived exosomes inhibit pyroptosis through promoting autophagy in LPS plus ATP-treated BV2 cells

**Fig. 4:**
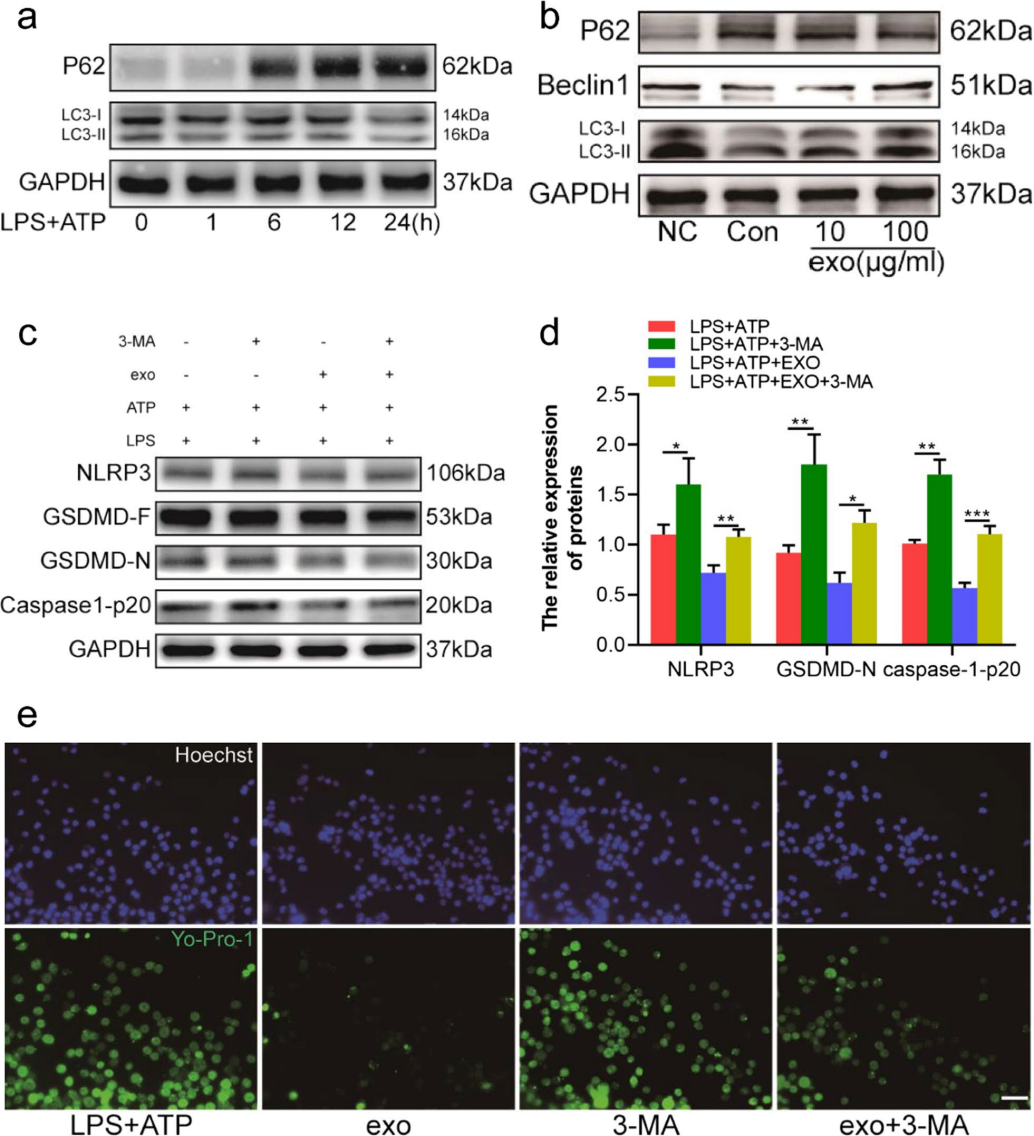
Inhibiting autophagy weakened the protective effect of huc-MSCs-derived exosomes on attenuating BV2 cells pyroptosis. **a** BV2 cells were administered LPS (1 µg/ml) for 1 h, 6 h, 12 h, 24 h and ATP (5 mM) for 30 min, and LC3, P62 were tested by western blot at the indicated time points. **b** Compared with con group, the protein levels of LC3-II and beclin1 were increased in 10 and 100 µg/ml exosomes pretreated BV2 cells, and the expression of P62 was inhibited. **c**, **d** Expression of NLRP3, GSDMD-F, GSDMD-N, caspase1-p20 was tested by western blot and subsequent quantitative analysis of the proteins as normalized to the LPS + ATP group. **e** BV2 cells pretreated with LPS, ATP, huc-MSCs-derived exosomes, 3-MA were stained with Yo-Pro-1 and Hoechst, bar = 50 µm. Data are presented as the mean ± standard deviation (SD). (*P < 0.05, **P < 0.01, ***P < *0.001*)

Abnormal autophagy aggravates inflammation and chronic pain [33]. It is elusive whether huc-MSCs-derived exosomes inhibit NLRP3 inflammasome activation via regulating the function of autophagy in inflammatory pain. First, we tested the levels of classic autophagy-related proteins in BV2 cells, pretreated with LPS for 0, 1, 6, 12 and 24 h and ATP for 30 min. The expression level of LC3-II was remarkably lower, while the level of P62 was upregulated with the extension of stimulation time as shown by western blot (Fig. 4a). The expression levels of autophagy-related proteins LC3, beclin1 and P62 were reversed in BV2 cells pretreated with 10 mg/mL and 100 mg/mL huc-MSCs-derived exosomes for 1 h, as measured by western blot (Fig. 4b). To further investigate the protective effect of huc-MSCs-derived exosomes on autophagy, TEM was used to observe changes in autophagosomes [34]. Our results showed that the amount of autophagosome increased in BV2 cells pretreated with 100 mg/mL huc-MSCs-derived exosomes (Additional file 1: Fig. S1). To investigate whether autophagy participates in the anti-inflammatory and anti-pyroptotic processes of huc-MSCs-derived exosomes, the autophagy inhibitor, 3-MA, was used to study the related mechanism. We found that the expression of NLRP3, caspase1-p20 and GSDMD-N was increased in BV2 cells pretreated with 3-MA compared with the control group. Furthermore, 3-MA weakened the inhibitory effect of huc-MSCs-derived exosomes on pyroptosis, as measured by Yo-Pro-1 staining and western blotting (Fig. 4c–e). In general, the results demonstrated that huc-MSCs-derived exosomes inhibited pyroptosis by promoting autophagy in LPS and ATP-treated BV2 cells.

### Huc-MSCs-derived exosomes regulate autophagy and pyroptosis via miR-146a-5p/TRAF6 in *vitro*

**Fig. 5:**
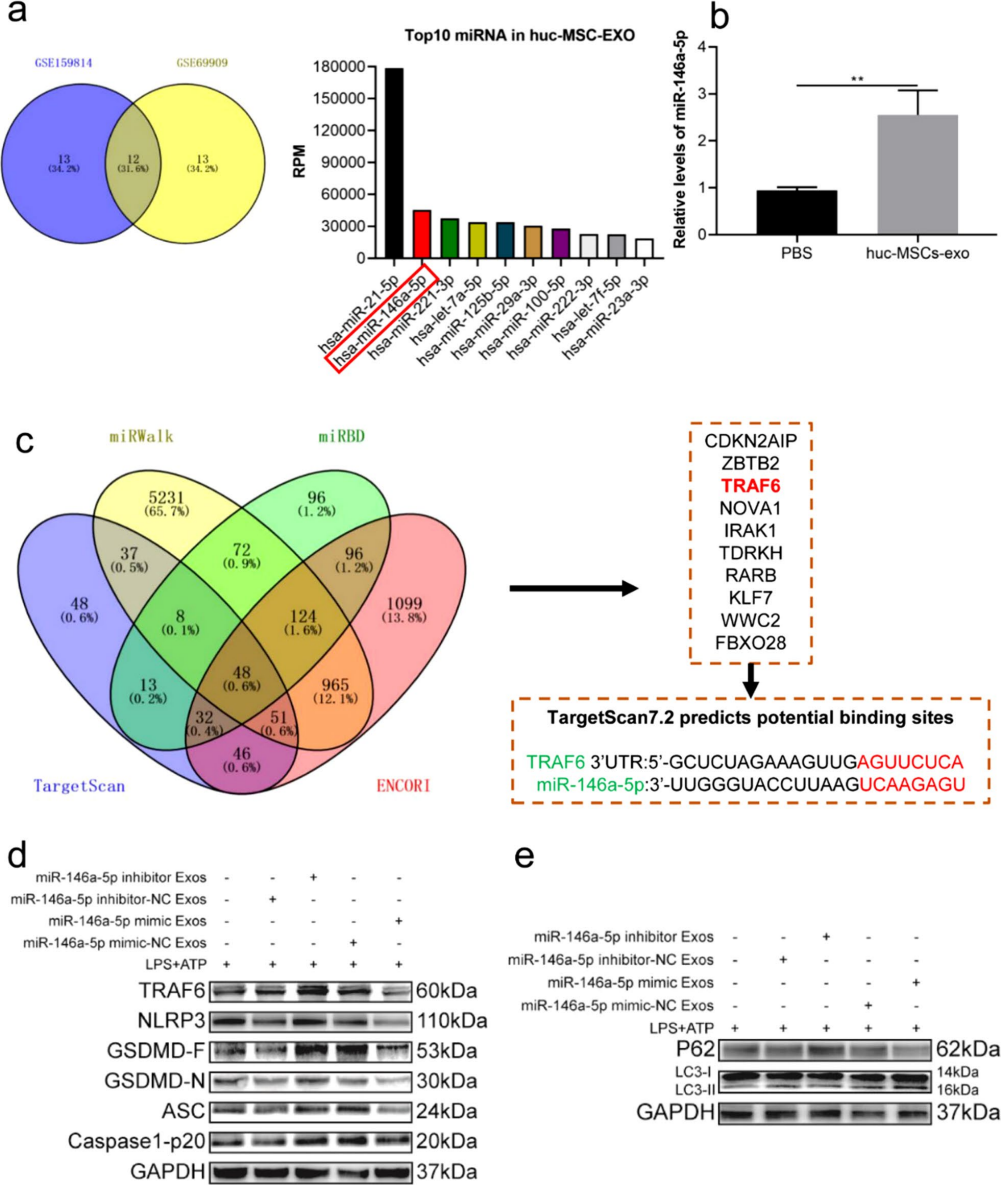
Huc-MSCs-derived exosomes inhibited pyroptosis and increased autophagy via transferring miR-146a-5p to BV2 cells. **a** Top ten miRNAs in GSE159814 and GSE69909. **b** RT-PCR was used to assay the relative levels of miR-146a-5p in BV2 cells treated with huc-MSCs-derived exosomes or PBS. **c** Four different miRNA target databases, miRWalk, miRBD, TargetScan, ENCORI, were used to predict the potential target genes of miR-146a-5p. **d** The protein levels of TRAF6, NLRP3, GSDMD-F, GSDMD-N, ASC and caspase1-p20 were measured by western blot in BV2 cells pretreated with miR-146a-5p inhibitor exosomes or miR-146a-5p mimic exosomes. **e** Autophagy-related proteins, P62 and LC3, were tested by western blot in BV2 cells pretreated with miR-146a-5p inhibitor exosomes or miR-146a-5p mimic exosomes. Data are presented as the mean ± standard deviation (SD). (**P < *0.01*)

miRNAs, one of the most important cargos in exosomes, are transferred to other cells and play important roles in regulating inflammatory diseases, such as spinal cord injury [35] and colitis [13]. Therefore, to investigate the possible mechanism by which huc-MSCs-derived exosomes influence autophagy and pyroptosis, we analysed the expression of miRNAs in datasets GSE159814 and GSE69909 [36, 37]. The performed analysis revealed the top 10 miRNAs in huc-MSCs-derived exosomes, and miR-146a-5p was in second place (Fig. 5a). Compared with that in the PBS group, the relative expression of miR-146a-5p in BV2 cells pretreated with huc-MSCs-derived exosomes was significantly increased as shown by RT-qPCR (Fig. 5b). Furthermore, huc-MSCs-derived exosomes labelled with PKH26 were incubated with BV2 cells for 24 h. Our results showed that PKH26- labelled exosomes were internalized by BV2 cells at 24 h (Additional file 1: Fig. S2). Lots of studies have demonstrated that miRNAs play their biological roles via repressing the expression and function of target genes [38]. Thus, we used the public databases miRWalk, miRBD, TargetScan, and ENCORI to predict the target genes of miR-146a-5p. There were 48 common genes in the four public databases, and TRAF6 was one of the most relevant target genes (Fig. 5c). The potential binding sites of miR-146a-5p and TRAF6 were predicted by TargetScan7.2 (http://www.targetscan.org/vert_72/) (Fig. 5c). To further verify the regulatory relationship between miR-146a-5p and TRAF6, huc-MSCs were transfected with miR-146a-5p mimic, inhibitor and negative control. Different groups of huc-MSCs-derived exosomes, miR-146a-5p mimic Exos, miR-146a-5p mimic-NC Exos, miR-146a-5p inhibitor Exos, and miR-146a-5p inhibitor-NC Exos, were used to pretreat BV2 cells. Results showed that the expression levels of TRAF6, NLRP3, ASC, caspase1-p20 and GSDMD-N were reduced in miR-146a-5p mimic Exos group, and the opposite results were showed in miR-146a-5p inhibitor Exos group by western blot (Fig. 5d). Autophagy-related proteins LC3-II and P62were tested via western blotting, and results demonstrated that miR-146a-5p mimic exosomes increased LC3-II expression and decreased P62 expression (Fig. 5e). Generally, huc-MSCs-derived exosomes promoted autophagy and alleviated pyroptosis in LPS and ATP-treated BV2 cells via miR-146a-5p/TRAF6.

### TRAF6 knockdown promotes autophagy and attenuates pyroptosis in LPS plus ATP-treated BV2 cells

**Fig. 6:**
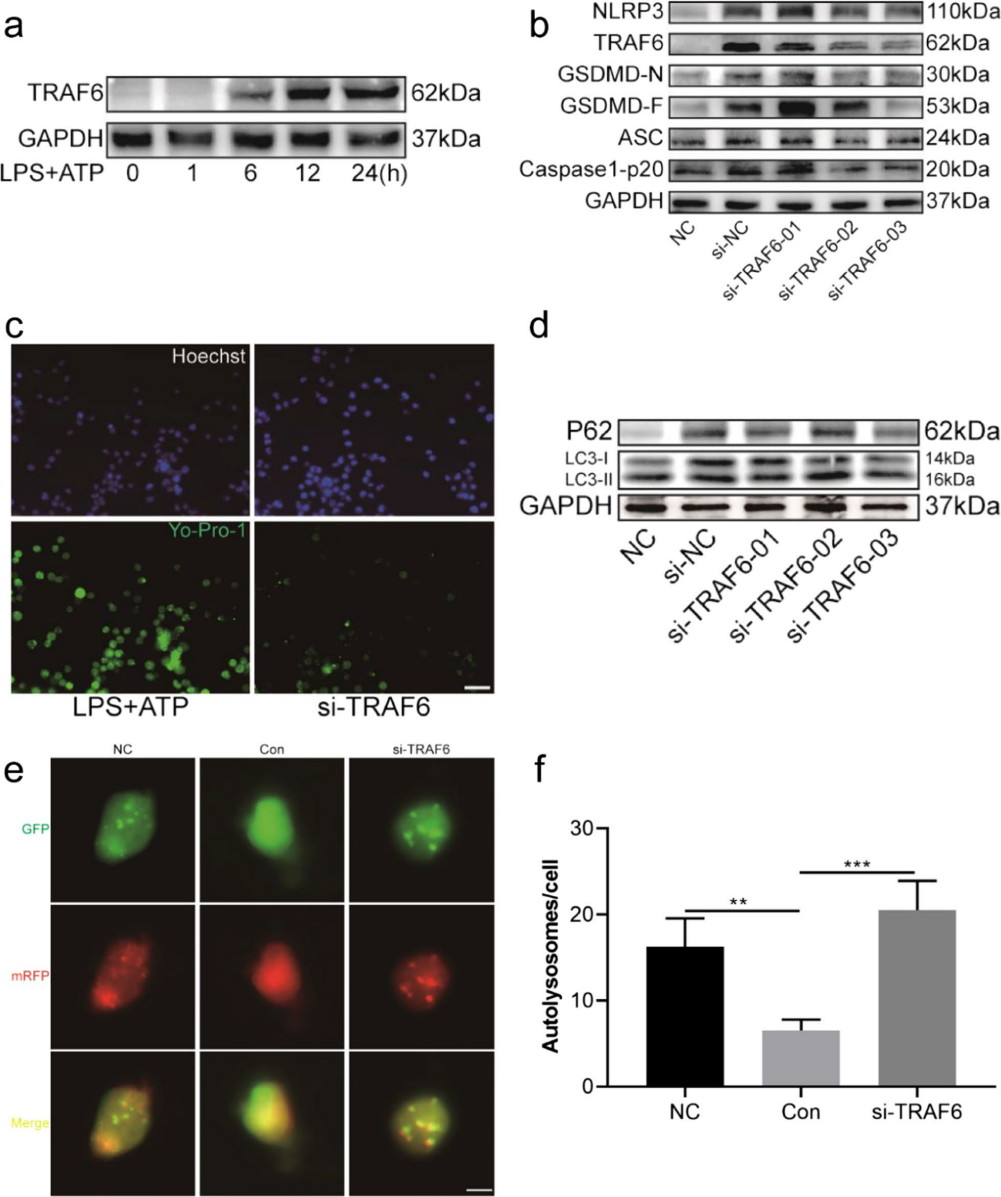
TRAF6 knockdown inhibited LPS and ATP induced BV2 cell pyroptosis and increased autophagy. **a** BV2 cells were administered LPS (1 µg/ml) for 0 h, 1 h, 6 h, 12 h, 24 h and ATP (5 mM) for 30 min, and TRAF6 was tested by western blot at the indicated time points. **b** Western blot analysis of NLRP3, TRAF6, GSDMD-F and GSDMD-N in BV2 cells transfected with siRNA targeting TRAF6 for 48 h and then stimulated with LPS (1 µg/mL) and ATP (5 mM) for 30 min. **c** BV2 cells pretreated with si-TRAF6, LPS, ATP were stained with Yo-Pro-1 and Hoechst, bar = 50 µm. **d** Levels of P62 and LC3 protein were measured by western blot in BV2 cells transfected with si-TRAF6. **e**, **f** Autophagy was visually observed via mCherry-GFP-LC3 adenovirus transfecting BV2 cells and quantitation of autolysosomes. Data are presented as the mean ± standard deviation (SD). (**P < *0.01*, ***P < *0.001*)

To measure the effect of TRAF6 on BV2 cell autophagy and pyroptosis, si-TRAF6 was transfected. TRAF6 protein expression was upregulated in LPS plus ATP-treated BV2 cells in a time-dependent manner (Fig. 6a). Western blot analysis showed that the expression levels of TRAF6, NLRP3, ASC, caspase1-p20 and GSDMD-N were inhibited in si-TRAF6 groups (Fig. 6b). Yo-Pro-1 dye was also used to observe the inhibitory effect of TRAF6 knockdown on pyroptosis (Fig. 6c). To investigate the protective value of si-TFAF6 in promoting autophagy, the western blot results were measured. The ratio of LC3-II/ LC3-I was increased in the si-TFAF6 group compared with the si-NC group, and the expression of P62 was inhibited in the si-TFAF6 group (Fig. 6d). To further confirm effects of si-TFAF6 on LPS plus ATP-induced changes in autophagy, BV2 cells were transfected with mCherry-GFP-LC3 adenovirus to observe autophagic flow as reported [28]. Results showed that LPS plus ATP stimulation triggered a dramatic decrease in autophagosomes (yellow puncta) and autolysosomes (red puncta) in BV2 cells, and si-TRAF6 transfection reversed the result with more autolysosomes (Fig. 6e, f). Overall, TRAF6 aggravated the activation of the NLRP3 inflammasome and GSDMD-induced pyroptosis, and played a negative role in autophagy.

### Huc-MSCs-derived exosomes promote autophagy and suppress pyroptosis by transferring miR-146a-5p to the spinal cord dorsal horn

**Fig. 7:**
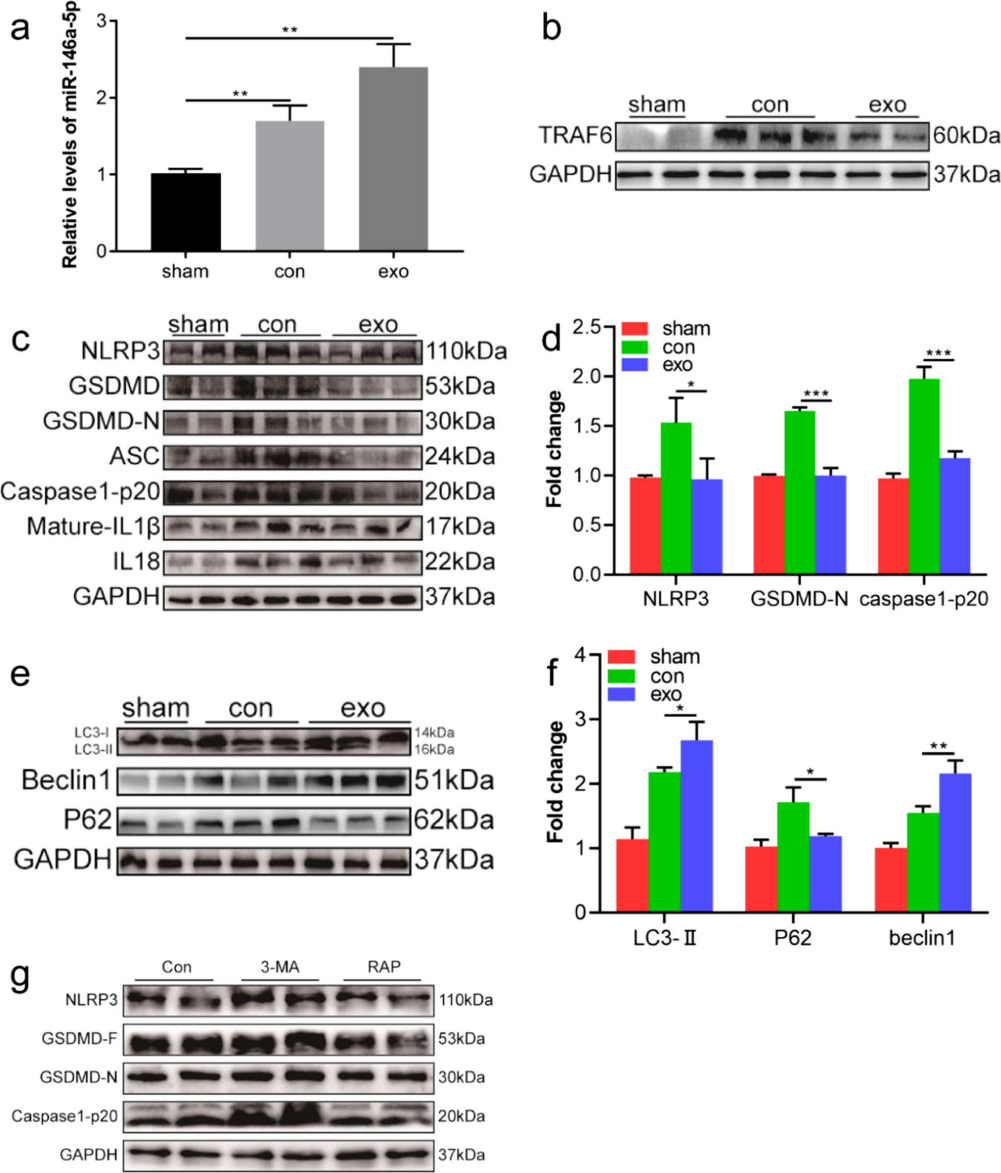
Huc-MSCs-derived exosomes inhibited pyroptosis through promoting autophagy via transferring miR-146a-5p in vivo. a The mRNA levels of miR-146a-5p in different groups were measured by RT-PCR. **b** Expression level of TRAF6 protein in the spinal cord measured by western blotting. **c**, **d** Huc-MSCs-derived exosomes decreased the expression of NLRP3 and pyroptosis-related proteins in the spinal cord. **e**, **f** Expression levels of LC3, beclin1, and P62 were measured in mice intrathecally injected with huc-MSCs-derived exosomes. **g** Mice were administered vehicle or 3-MA, RAP via intrathecal injection 1 h before modelling, and NLRP3, GSDMD-F, GSDMD-N, and Caspase1-p20 proteins were tested by western blot. Data are presented as the mean ± standard deviation (SD). (*P < 0.05, **P < 0.01, ***P < *0.001*)

To explore the mechanism by which huc-MSCs-derived exosomes attenuate CFA-induced inflammatory pain in vivo, mice were intrathecally injected with huc-MSCs-derived exosomes for 4 consecutive days. The results confirmed that the expression level of miR-146a-5p was increased in the spinal cord dorsal horn after huc-MSCs-derived exosomes injection by RT-qPCR (Fig. 7a). Furthermore, the expression level of TRAF6 was markedly reduced in the spinal cord dorsal horn of mice injected with huc-MSCs-derived exosomes (Fig. 7b). Importantly, the expression levels of NLRP3, ASC, caspase1-p20, mature IL-1β, IL-18, and GSDMD-N were markedly decreased in the exosomes group compared with the control group (Fig. 7c, d). On the 3rd day, autophagy-related proteins, LC3-II and beclin1, were upregulated after pretreatment with huc-MSCs-derived exosomes. However, the expression level of P62 was repressed (Fig. 7e, f). 3-MA and RAP were intrathecally injected 1 h before modelling to explore the relationship between autophagy and pyroptosis in CFA-induced inflammatory pain. Results demonstrated that 3-MA increased the levels of NLRP3, caspase1-p20, and GSDMD-N via western blotting. The result of the RAP group, an inducer of autophagy, was the opposite (Fig. 7g). In general, these data demonstrated that huc-MSCs-derived exosomes promoted autophagy which alleviated pyroptosis in CFA-induced inflammatory pain by transferring miR-146a-5p to the spinal cord dorsal horn.

## Discussion

Aberantly activated microglia, an innate immune cell, aggravate inflammatory pain by upregulating inflammatory factors [39, 40]. Thus, intensive investigations have shown that NLRP3 inflammasome activation triggers functional changes in microglia, which are involved in the initiation and development of chronic pain [12, 41]. Microglia pyroptosis is a newly reported inflammatory cell death that occurs through the NLRP3 inflammasome. However, it is unclear whether microglia pyroptosis participates in inflammatory pain via NLRP3/GSDMD. A number of studies have been performed to estimate the role that inflammasomes play in inflammatory pain. It is still controversial whether the NLRP3 inflammasome contributes to inflammatory pain. In acute inflammatory pain induced by carrageenan, the NLRC4 inflammasome, not the NLRP3 inflammasome, is involved in the peripheral activation of caspase-1 and IL-1β maturation, which participates in the genesis of hyperalgesia and neuroinflammation [42]. Another study demonstrated that the activation of the NLRP2 inflammasome increased the activation of DRG neurons and the subsequent development of inflammatory pain hypersensitivity [43]. On the other hand, amounts of investigations have reported the function of the NLRP3 inflammasome in inflammatory pain. One study showed that NLRP3^KO^ male mice were protected from surgery-induced postoperative inflammation and neuronal sensitization-induced postoperative inflammatory pain [44]. Furthermore, CFA injection induces the activation of the NLRP3 inflammasome in paw skin macrophages, which promotes the maturation of the inflammatory cytokine IL-1β via cleaved pro-caspase1 [45]. The differences between these studies may be attributed to the different inflammatory models and sampling sites. To measure the role of the NLRP3 inflammasome and GSDMD-induced pyroptosis in central nervous system, we pay more attention to the related mechanism in the spinal cord. Our results are consistent with previous studies, in which the protein levels of NLRP3, ASC, caspase1-p20, GSDMD and GSDMD-N substantially increased during inflammatory pain. Immunofluorescence staining showed that caspase1-p20 and GSDMD-N were mainly colocalized with iba1-positive microglia in the spinal cord (Additional file 1: Fig. S3). Thus, our data suggest that inhibiting NLRP3/GSDMD-induced microglial pyroptosis is a new therapeutic strategy for inflammatory pain.

Autophagy serves as another defensive means that has been implicated in repressing neuroinflammation and chronic pain via inhibiting NLRP3 inflammasome activation [15, 46]. However, the exact regulatory mechanism between autophagy and pyroptosis in CFA-induced inflammatory pain is still elusive. Autophagy plays an important role in eliminating misfolded proteins, ROS, ATP and proinflammatory cytokines which induces NLRP3 inflammasome activation [47]. What's more, increasing evidence has shown that autophagy-related proteins can package and degrade the NLRP3 inflammasome components ASC and NLRP3 [48]. Importantly, autophagy can also directly inhibit the expression of pro-IL-1β and eliminate mature IL-1β [49]. Gouty arthritis (GA) is a common inflammatory disease with persistent inflammatory pain as a result of macrophage pyroptosis. Mitophagy is promoted by triggering the Pink1/Parkin pathway, which significantly improves GA by inhibiting the pyroptosis of macrophages [50]. Spinal cord injury (SCI) is a common central nervous system inflammatory disease that often results in disabling motor and sensory functions. Studies have investigated its underlying mechanisms, revealing that promoting autophagy and inhibiting pyroptosis via the AMPK-mTOR-TFEB signalling pathway improve the function of spinal cord after SCI [51]. In our previous study, we found that the dysfunction of autophagy in astrocytes aggravated mechanical allodynia and thermal hyperalgesia by promoting the release of proinflammatory cytokines [15]. It is an urgent problem to clarify the pathogenesis and seek effective treatments for chronic pain. In the present study, we report for the first time that microglia pyroptosis inhibited by autophagy plays an important role in CFA-induced inflammatory pain. Our data also showed that intrathecal injection of huc-MSCs-derived exosomes relieved central inflammation in mice with chronic inflammatory pain. Further analysis demonstrated that huc-MSCs-derived exosomes promoted autophagy and inhibited NLRP3/GSDMD-induced pyroptosis in the spinal cord dorsal horn.

**Fig. 8:**
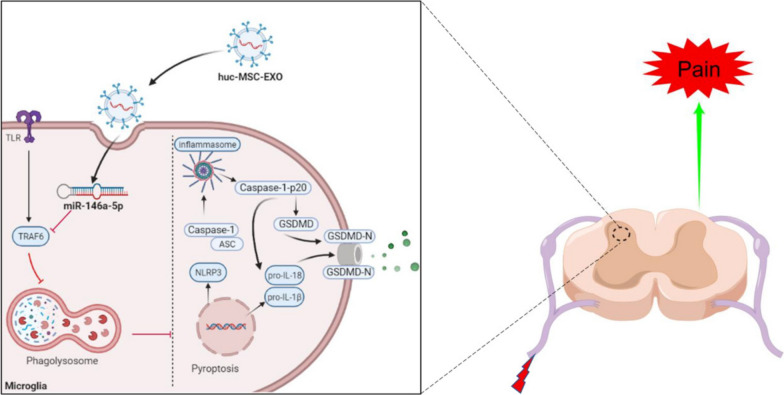
Proposed mechanism: huc-MSCs-derived exosomes inhibit pyroptosis through promoting autophagy via miR-146a-5p/TRAF6 in CFA-induced inflammatory pain

Amounts of studies have confirmed the protective value of MSCs in chronic pain, including neuropathic pain, bone cancer pain and inflammatory pain [19, 52]. The core mechanism in this process is paracrine rather than MSCs proliferation and differentiation. Exosomes have been the most studied paracrine substance during the past few years and are widely used as a treatment strategy for inflammatory diseases, such as neuropathic pain [22], spinal cord injury [35], and Parkinson’s disease [53]. A few unique advantages make it possible for MSC-derived exosomes to replace MSCs in central nervous system diseases. Exosomes, as lipid nanovesicles, are able to directly pass through blood brain barrier and easily reach lesion sites [22]. Exosomes play critical roles in cell-to-cell communication by transferring RNA, proteins, DNA and lipids. miRNAs are important cargos transferred by exosomes which inhibit target genes expression by restraining mRNA translation and accelerating mRNA degradation [54]. Inflammatory bowel disease, a complex inflammatory disorder, can be relieved by huc-MSCs-derived exosomes, which inhibit NLRP3 inflammasomes and cell pyroptosis through transferring miR-378a-5p [13]. Exosomes protect SCI from glia scarring and axonal injury by transferring miR-26a to the spinal cord [35]. Our data also demonstrated that the miR-146a-5p enriched in huc-MSCs-derived exosomes promoted microglia autophagy and inhibited microglia pyroptosis by modulating its target gene TRAF6 (Fig. 8). Increasing evidence has proven the protective value of miR-146a-5p in chronic pain [55, 56]. In the animal model of stroke, huc-MSCs-derived exosomes inhibit the microglia-mediated inflammatory response and promote neuronal repair via miR-146a-5p/TRAF6 axis [57]. TRAF6, as an E3 ubiquitin ligase, participates in many inflammatory diseases. TRAF6, which is expressed in astrocytes, aggravates SNL-induced neuropathic pain by increasing CCL2 secretion [58]. Our data showed that TRAF6 inhibited autophagy and increased microglia pyroptosis in CFA-induced inflammatory pain.

## Conclusion

Generally, the present study provides evidence that huc-MSCs-derived exosomes effectively alleviate the activation of the NLRP3 inflammasome and GSDMD-induced microglia pyroptosis by promoting autophagy in CFA-induced chronic inflammatory pain. The process is regulated by TRAF6, which is inhibited by miR-146a-5p enriched in huc-MSCs-derived exosomes. These data indicate that huc-MSCs-derived exosome is a promising treatment strategy for chronic inflammatory pain.

## Supplementary Information

**Additional file 1: Figure S1.** Huc-MSCs-derived exosomes increased the number of autophagosomes in BV2 treated with LPS and ATP. **Figure S2**. Huc-MSCs-derived exosomes were internalized by BV2 cells. **Figure S3**. Double immunofluorescence staining of NLRP3, caspase1-p20 and GSDMD with iba1.

## Data Availability

All data and marerials are showed in the paper and further inquiries can be directed to the corresponding author.

## Abbreviations

TRAF6: TNF receptor associated factor 6
NLRP3: NOD-like receptor thermal protein domain associated protein 3
IL-1β: Interleukin-1β
3-MA: 3-Methyladenine
DSS: Dextran sulfate sodium
LC3: Microtubule-associated protein 1 light3
TBST: Tris-Buffered Saline and Tween
SNL: Spinal nerve ligation
CCL-2: C–C motif ligand 2
iba1: Ionized calcium binding adapter molecule 1
