# Supplementary material for: Huc-MSCs-derived exosomes attenuate inflammatory pain by regulating microglia pyroptosis and autophagy via the miR-146a-5p/TRAF6 axis
Source: J Nanobiotechnology. 2022 Jul 14;20:324. doi: 10.1186/s12951-022-01522-6 (PMC9281091; doi:10.1186/s12951-022-01522-6)
Supplement: Supplementary file 1 — Additional file 1: Figure S1. Huc-MSCs-derived exosomes increased the number of autophagosomes in BV2 treated with LPS and ATP. Figure S2. Huc-MSCs-derived exosomes were internalized by BV2 cells. Figure S3. Double immunofluorescence staining of NLRP3, caspase1-p20 and GSDMD with iba1. [file 12951_2022_1522_MOESM1_ESM.docx]

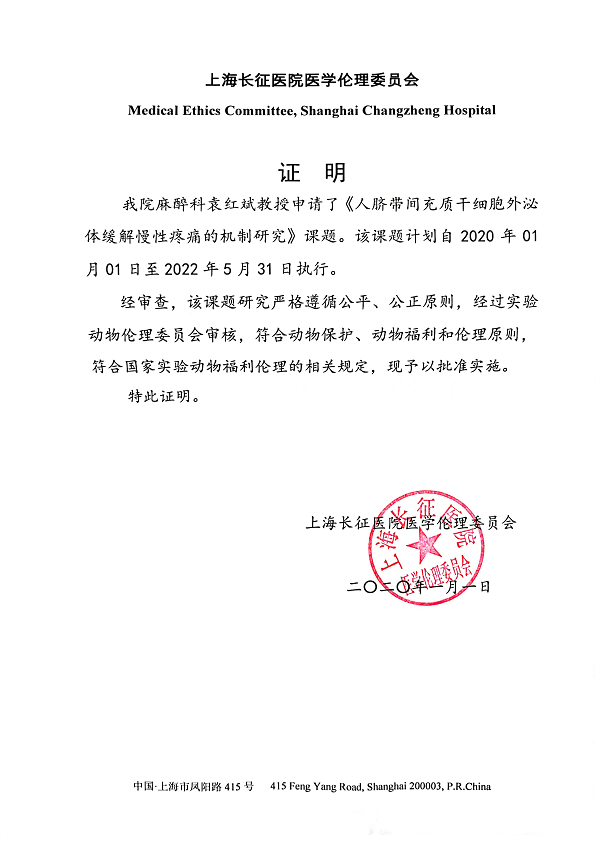


**Fig. s1:** Animal ethics certificate.


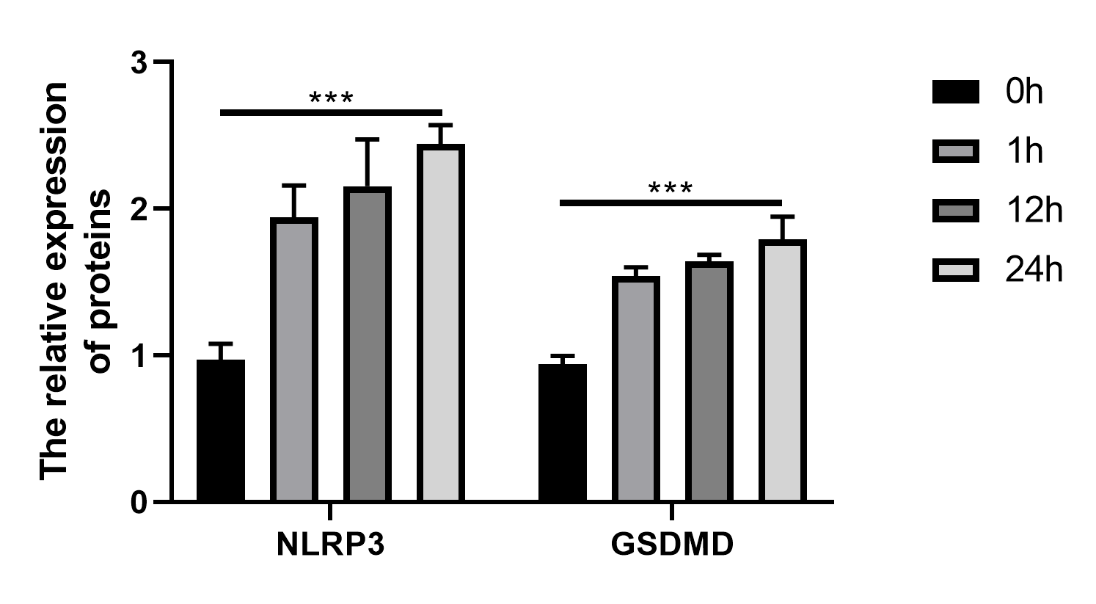


**Fig. s2:** Subsequent quantitative analysis of the proteins as normalized to the 0 h group. (**** P<0.001*)


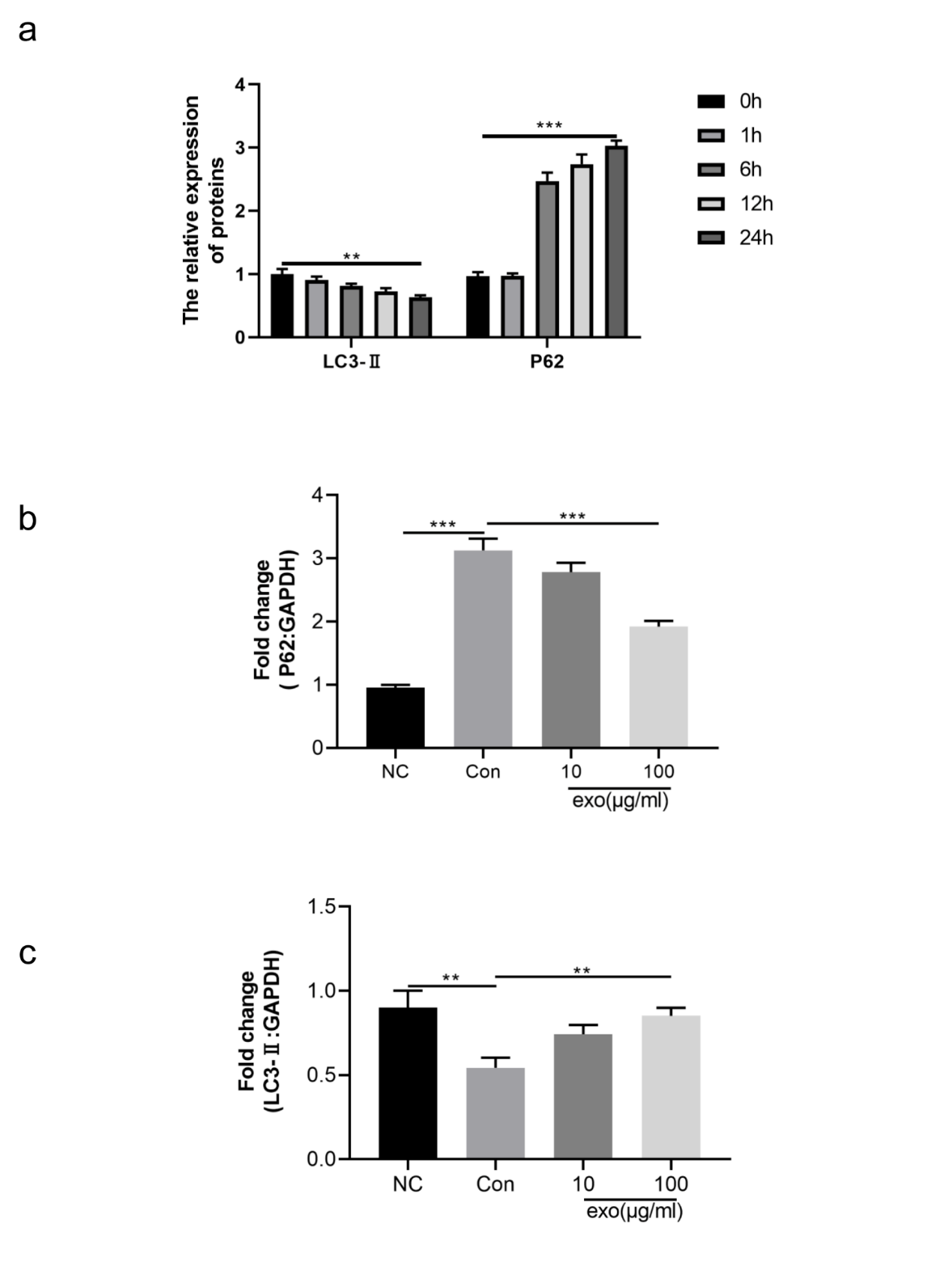


**Fig. s3: a** Subsequent quantitative analysis of the proteins as normalized to the 0 h group. b Subsequent quantitative analysis of P62 as normalized to the NC group. c Subsequent quantitative analysis of LC3-Ⅱ as normalized to the NC group. (*** P<0.01,*** P<0.001*)


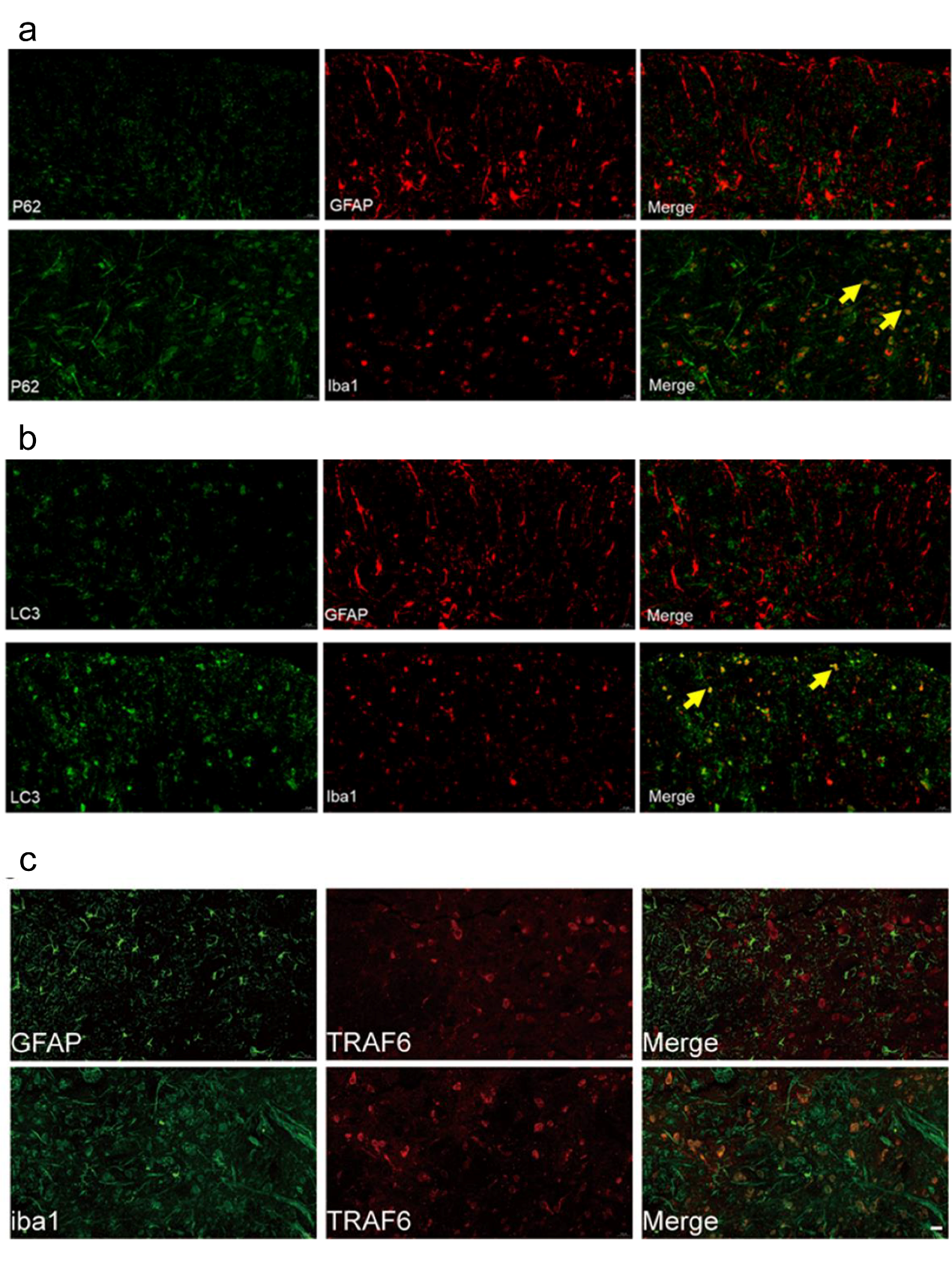


**Fig. s4: a** Double immunofluorescence staining of P62 with microglia marker iba1, bar=20 µm. b Double immunofluorescence staining of LC3 with microglia marker iba1, bar=20 µm. c Double immunofluorescence staining of TRAF6 with microglia marker iba1, bar=20 µm.
